# Supplementary material for: Imagined speech event detection from electrocorticography and its transfer between speech modes and subjects
Source: Commun Biol. 2024 Jul 5;7:818. doi: 10.1038/s42003-024-06518-6 (PMC11226700; doi:10.1038/s42003-024-06518-6)
Supplement: Supplementary file 2 — Supplementary material [file 42003_2024_6518_MOESM2_ESM.pdf]

Imagined speech event detection from electrocorticography and its transfer between speech modes and subjects

Supplementary material

Supplement to Methods section

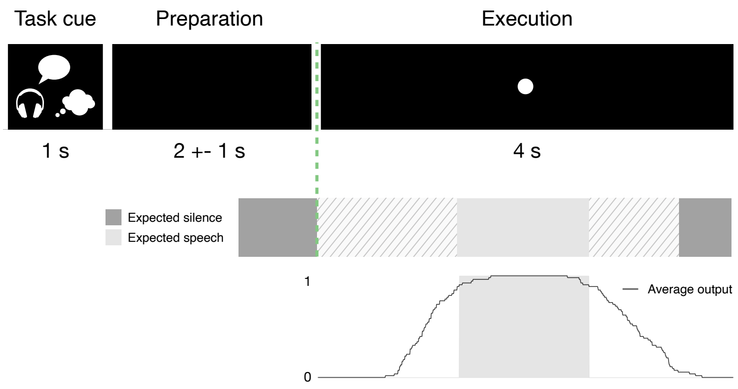

*Supplementary Figure 1 – **Imagined speech timing**. The upper part of the figure shows an excerpt of the paradigm timing. The middle part shows the surrogate labels for imagined speech for participant P9, dark grey indicates windows labelled as 'no speech', light grey the window labelled as 'speech' and the hatched pattern the windows that are discarded (see Methods section for an explanation). The lower part depicts the average output of performed speech trials for the same subject (0/1 indicates the absence/presence of speech, respectively). The grey shaded area delimits the window for which the average output surpasses the 0.9 threshold.*

	Age	Gender	Handedness	Speech lateralization	Number of trials		
					Speaking	Listening	Imagining
P1	26	M	R	L	59	59	58
P2	47	M	R	R	43	59	57
P3	51	F	R	Left language dominance for expressive language, no dominance for the receptive language	53	60	57
P4	24	F	R (Left footed)	Left language dominance, bilateral language representation	57	60	60
P5	52	F	ambidextrous	L	58	60	60
P6	33	F	R	L	59	60	60
P7	24	F	R	No clear speech lateralization	54	60	60
P8	18	F	R	Left for expressive language, right for receptive language	56	60	59
P9	42	M	R	L	57	60	58
P10	47	F	R	Left for language production and reading, with only limited activation in the right hemisphere	51	59	57
P11	55	M	R	-	59	60	60
P12	23	F	R	-	55	60	60
P13	20	F	R	L	60	60	60
P14	38	F	R	L	52	60	53
P15	21	F	L	Left for expressive language, no result for receptive language	49	0	120
P16	22	F	L (but also does things right, e.g., playing tennis)	L	57	60	60

*Supplementary Table 1 – **Clinical information.** L = left, R = right, F = female, M = male*

Morgen gaan we naar de stad	*Tomorrow, we will go to the city*	De dader werd vervolgd	*The perpetrator was prosecuted*
Ze klopten op de deur	*They knock on the door*	De kaartjes zijn gratis	*The tickets are free*
De leraar deelde straf uit	*The teacher handed out punishment*	Het contract is verlengd	*The contract is extended*
De prijzen werden verlaagd	*Prices were lowered*	Het huis is niet bewoond	*The house is not inhabited*
Het interview was erg goed	*The interview was very good*	Sporten is erg gezond	*Exercising is very healthy*
Juwelen zijn erg kostbaar	*Jewelry is very expensive*	De muren zijn wit geschilderd	*The walls are painted white*
De reis is geannuleerd	*The trip has been cancelled*	De tafel is gedekt	*The table is set*
De man heeft een lange baard	*The man has a long beard*	Die acteur is heel bekend	*That actor is very famous*
De dief is opgespoord	*The thief is tracked down*	Zijn invloed is erg groot	*His influence is very great*
Zijn gedrag werd beloond	*His behavior was rewarded*	De straten zijn schoongeveegd	*The streets are cleaned up*

*Supplementary Table 2 – **List of sentences.** An English translation is provided for each sentence.*

	Speaking	Listening
P1	6.19e-01	1.02e-01
P2	9.36e-01	2.81e-01
P3	8.45e-01	1.68e-01
P4	7.15e-01	7.51e-01
P5	5.57e-01	8.82e-01
P6	8.67e-01	3.81e-01
P7	2.90e-01	8.84e-01
P8	2.78e-01	7.65e-01
P9	1.87e-02* / 7.37e-01	9.91e-01
P10	6.29e-01	3.04e-01
P11	1.45e-01	9.98e-01
P12	4.67e-01	6.72e-01
P13	6.18e-01	5.92e-01
P14	5.49e-01	1.37e-01
P15	3.67e-01	-
P16	2.97e-01	9.09e-01

*Supplementary Table 3 – **Assessment of the presence or absence of acoustic contamination**. The risk to wrongly consider the presence of contamination (P-value), estimated using the procedure developed by Roussel et al. (2020), is listed per subject for speaking and listening. The estimates are based on the 0-128 Hz range of which the upper limit is determined by the 256 Hz sampling rate. The results show no evidence of contamination except for subject P9 in which case the P-value was below 0.05 for performed speech only (marked with an asterisk). However, the same P-value exceeds 0.05 when based on the 75-128 Hz range in accordance with the argumentation of Roussel et al. (2020) that below 75 Hz no speech-related contamination is expected and that the outcome might be influenced by the 50 Hz line. Based on this, also the data of subject P9 was retained in our analysis. Note that no acoustic contamination is expected for imagined speech.*

Supplement to Figure 2

Description of Supplementary Fig. P1-16

For each participant, the individual brain model is shown with the implanted electrodes labeled by their accuracies per speech mode and the best electrode per speech mode labeled by the dotted lines (same color code as in Fig.2). Asterisks indicate the significance of the permutation test (* p<0.01, ** p<0.001, for p-values see Supplementary Table 4). When the accuracy on the passive dataset was not significantly above chance level, the accuracy (on the passive dataset) is indicated with a dot as an icon (e.g., see Fig. P4). Next to each brain model, the functional mapping is displayed, when available, as not for all patients functional mapping was clinically relevant. The circles filled in red, blue or green, or a combination thereof, indicate the best electrodes for performed, perceived and imagined speech, respectively. Circles with light blue and dark blue circumferences indicate electrodes with interictal activity at level 2 and 3, respectively (all other channels are considered level 1). D = dorsal, V = ventral, A = anterior, P = posterior, M = medial, R = right, L = left.

Participant 1

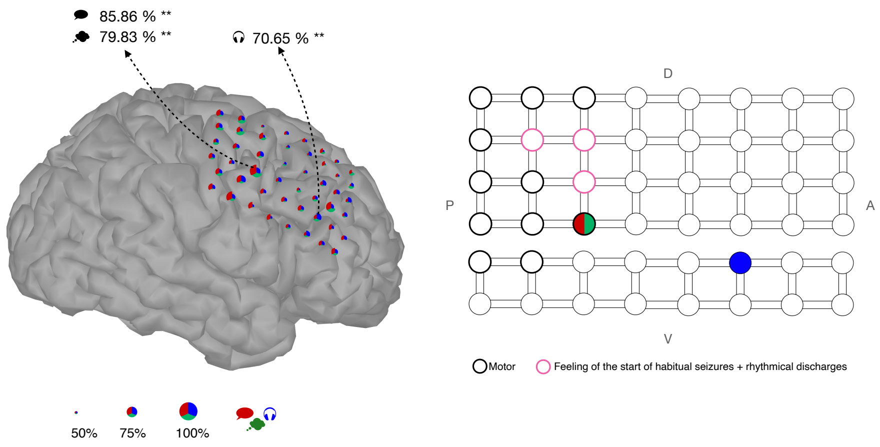

Supplementary Figure P1

Participant 2

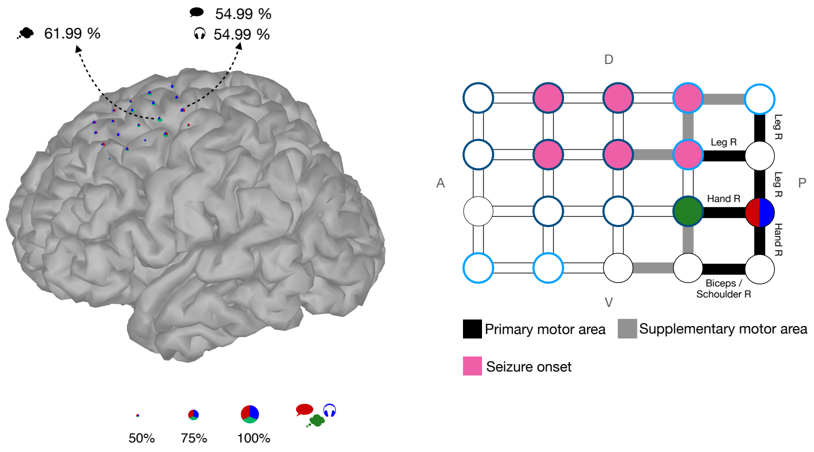

Supplementary Figure P2

Participant 3

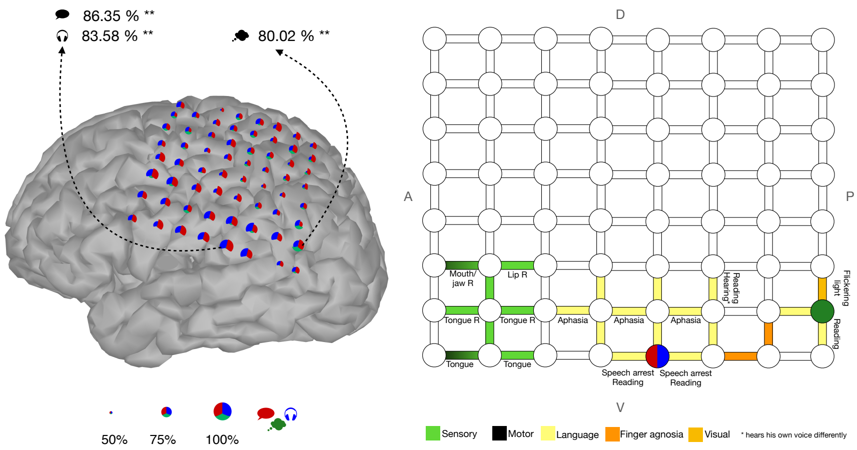

Supplementary Figure P3

Participant 4

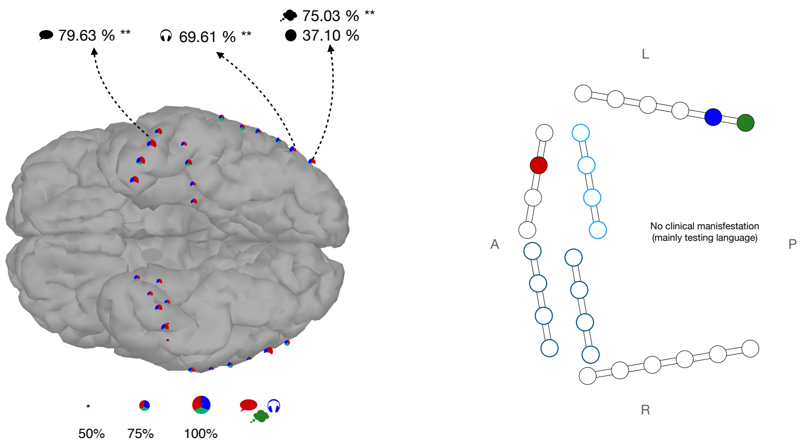

Supplementary Figure P4

Participant 5

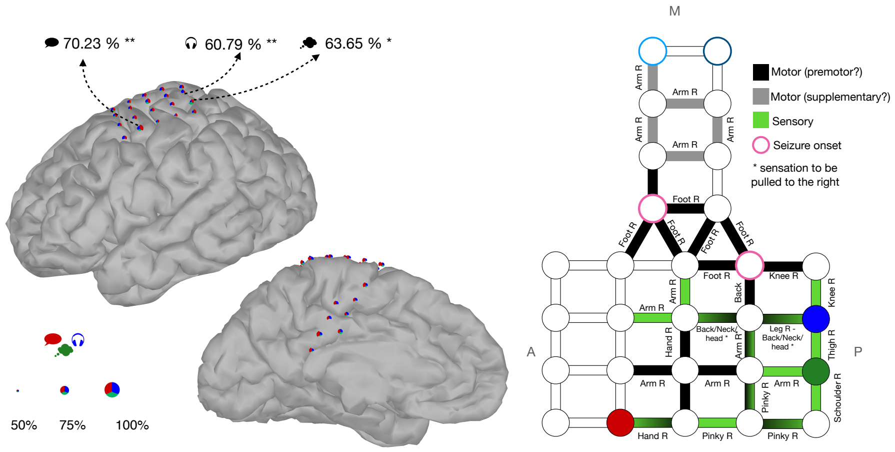

Supplementary Figure P5

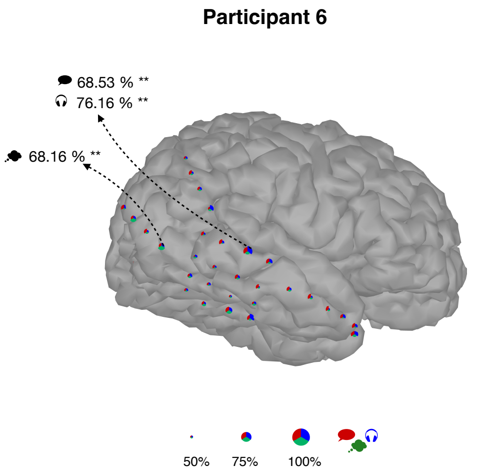

Supplementary Figure P6

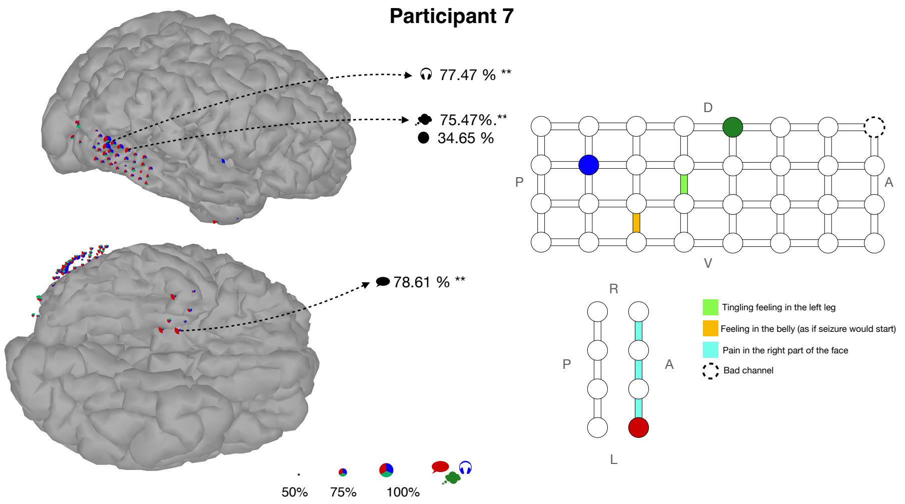

Supplementary Figure P7

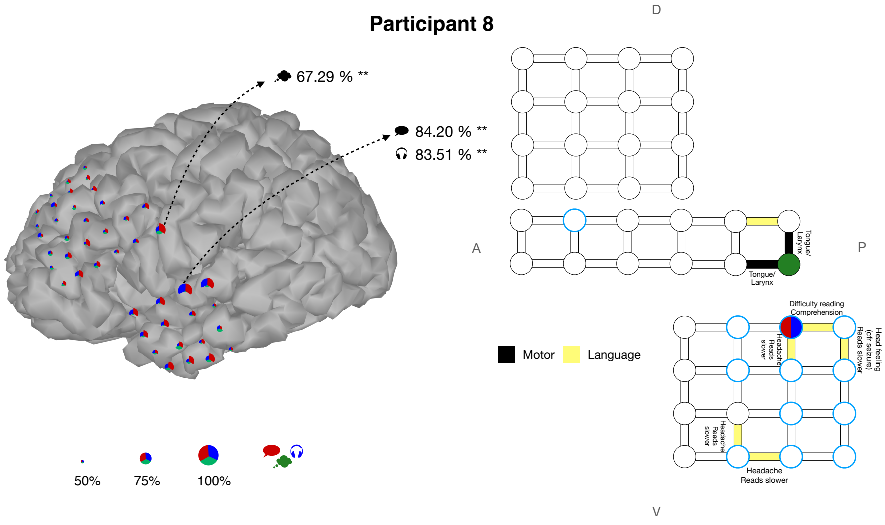

Supplementary Figure P8

Participant 9

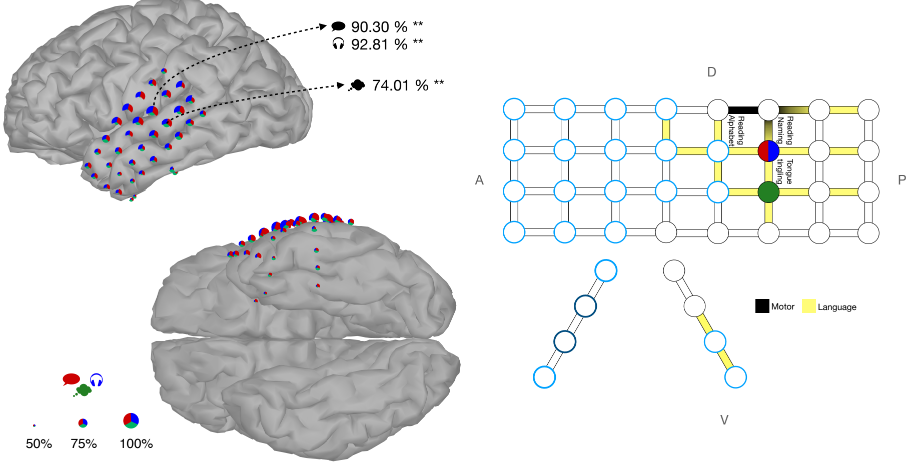

Supplementary Figure P9

Participant 10

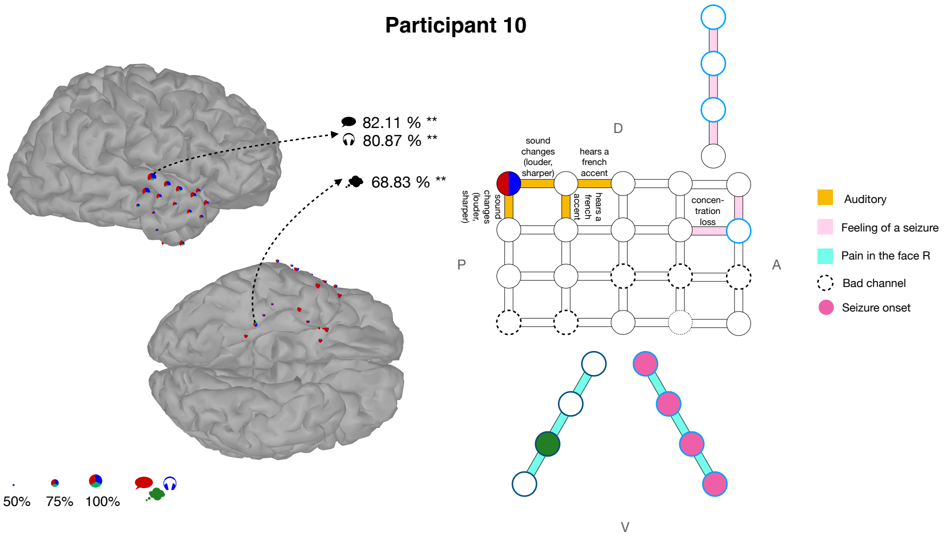

Supplementary Figure P10

Participant 11

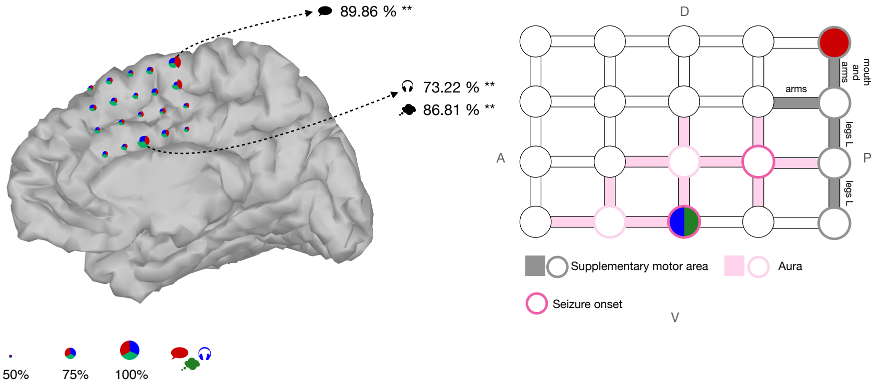

Supplementary Figure P11

Participant 12

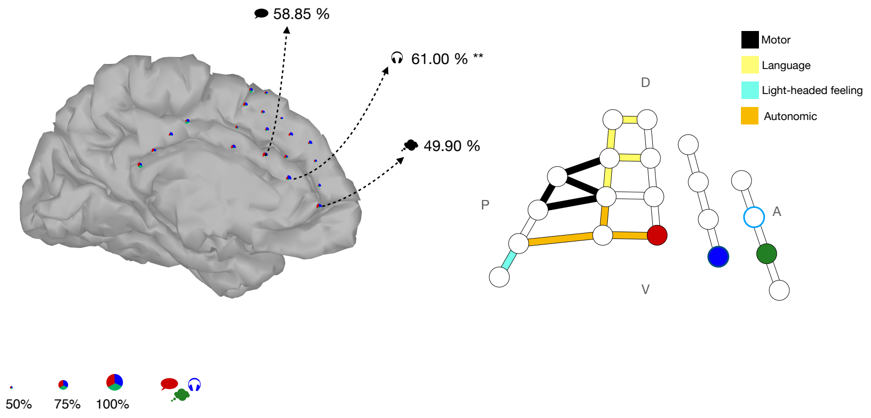

Supplementary Figure P12

Participant 13

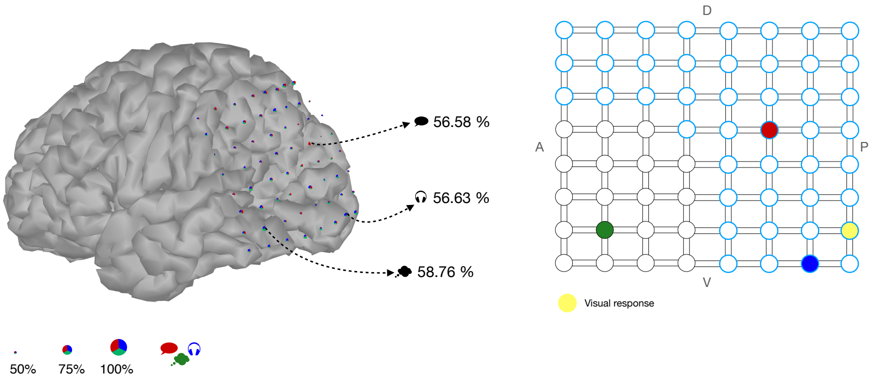

Supplementary Figure P13

Participant 14

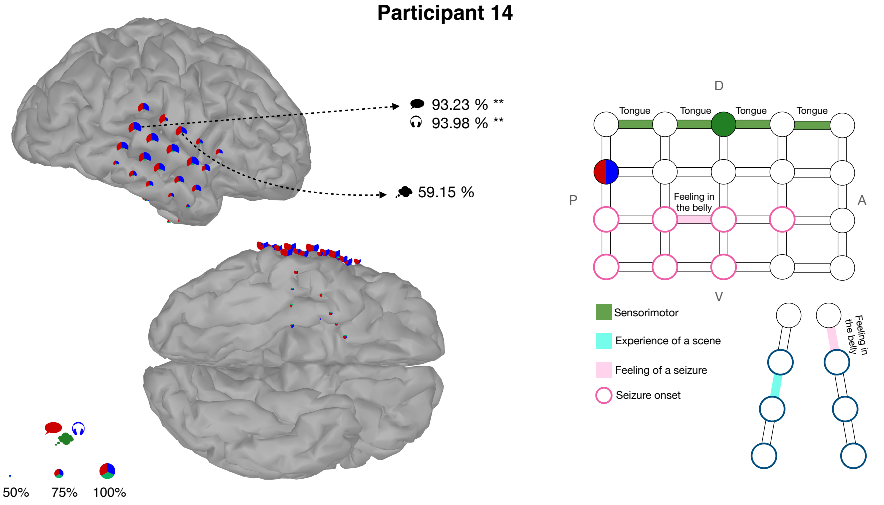

Supplementary Figure P14

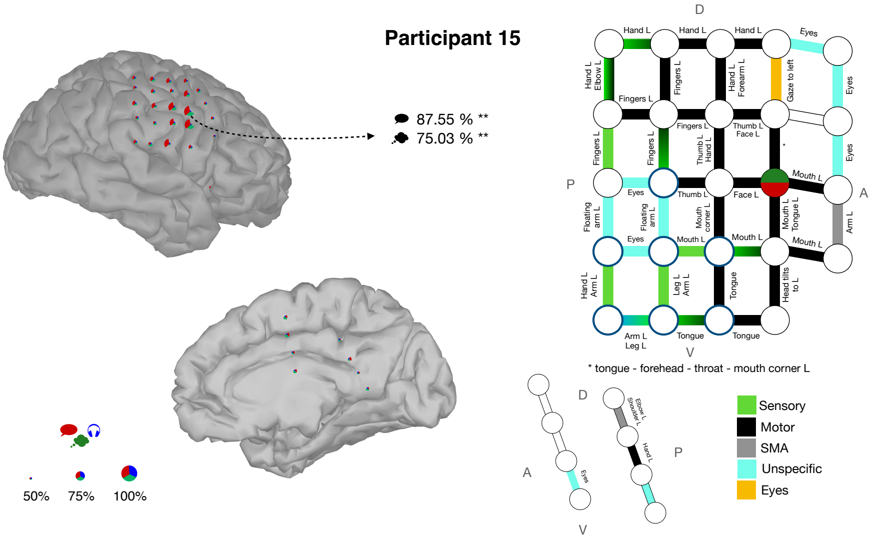

Supplementary Figure P15

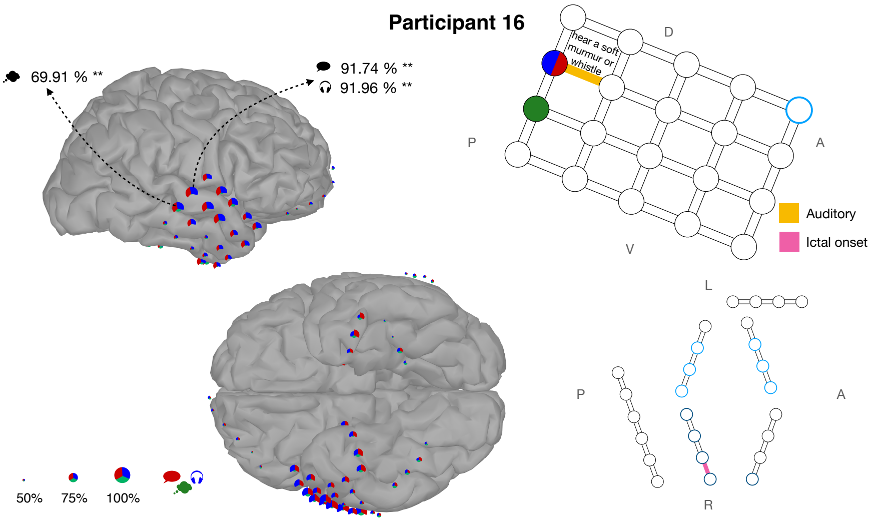

Supplementary Figure P16

	Best electrode for speaking		Best electrode for listening		Best electrode for imagining							
	Speaking		Listening		Imagining (active)		Imagining (passive)		Speaking		Listening	
	Accuracy	p-value	Accuracy	p-value	Accuracy	p-value	Accuracy	p-value	Accuracy	p-value	Accuracy	p-value
**P1**	85.86%	1.00e-04	70.65%	1.00e-04	79.83%	1.00e-04	67.43%	1.00e-04	same as best electrode for speaking		66.47%	1.00e-04
P2	54.99%	9.54e-02	54.99%	7.78e-02	61.99%	1.23e-02	/	/	/	/	/	/
**P3**	86.35%	1.00e-04	83.58%	1.00e-04	80.02%	1.00e-04	69.48%	1.00e-04	79.26%	1.00e-04	71.93%	1.00e-04
P4	79.63%	1.00e-04	69.61%	1.00e-04	75.03%	1.00e-04	37.10%	1.00e+00	/	/	/	/
**P5**	70.23%	1.00e-04	60.79%	6.00e-04	63.65%	3.00e-03	67.92%	1.00e-04	60.26%	2.40e-03	51.54%	3.45e-01
**P6**	68.53%	1.00e-04	76.16%	1.00e-04	68.16%	2.00e-04	62.83%	1.00e-04	58.64%	6.20e-03	62.82%	1.00e-04
P7	87.74%	1.00e-04	90.72%	1.00e-04	69.02%	1.00e-04	34.65%	1.00e+00	/	/	/	/
**P8**	84.20%	1.00e-04	83.51%	1.00e-04	67.29%	3.00e-04	72.99%	1.00e-04	81.62%	1.00e-04	63.59%	1.00e-04
**P9**	90.30%	1.00e-04	92.81%	1.00e-04	74.01%	1.00e-04	64.25%	1.00e-04	85.41%	1.00e-04	83.54%	1.00e-04
**P10**	71.19%	1.00e-04	65.58%	1.00e-04	71.87%	4.00e-04	62.35%	2.00e-04	58.16%	1.84e-02	61.96%	3.00e-04
**P11**	89.86%	1.00e-04	73.22%	1.00e-04	86.81%	1.00e-04	84.45%	1.00e-04	79.27%	1.00e-04	same as best electrode for listening	
P12	58.85%	1.05e-02	61.00%	4.00e-04	61.69%	1.14e-02	/	/	/	/	/	/
P13	56.58%	1.90e-02	56.63%	2.34e-02	58.76%	5.43e-02	/	/	/	/	/	/
P14	93.23%	1.00e-04	93.98%	1.00e-04	59.15%	3.41e-02	/	/	/	/	/	/
**P15**	87.55%	1.00e-04	-	-	75.03%	1.00e-04	75.69%	1.00e-04	same as best electrode for speaking		-	-
**P16**	91.74%	1.00e-04	91.96%	1.00e-04	69.91%	1.00e-04	63.07%	2.00e-04	89.78%	1.00e-04	89.01%	1.00e-04
Mean	78.55%		75.01%		70.14%							

*Supplementary Table 4 – **Performance of the best electrodes.** Accuracies and p-values are reported for the best performing electrodes for each speech mode. For the best imagined speech detection electrode, the performance is given for the active and passive datasets as well as for the other speech modes. Participants in bold font were included in the analyses mentioned in the subsection "Comparison between speech modes" of the Results section.*

	Number of electrodes					
	Left hemisphere			Right hemisphere		
Total	307			281		
Temporal	98			140		
	Speaking		Listening		Imagining	
	Left	Right	Left	Right	Left	Right
> 65 %	46	60	34	40	15	5
> 70 %	32	43	21	30	4	0

*Supplementary Table 5 – **Electrode count**. The total number of electrodes and the number of electrodes on temporal lobes (right and left hemispheres) are indicated. The last two rows indicate the number of electrodes on temporal lobes for which the accuracy exceeds 65 and 70 % for both the right and left hemispheres. For imagined speech, the performance for the active dataset is used, but the performance was set to 50% if the performance on the passive dataset was lower than 50%.*

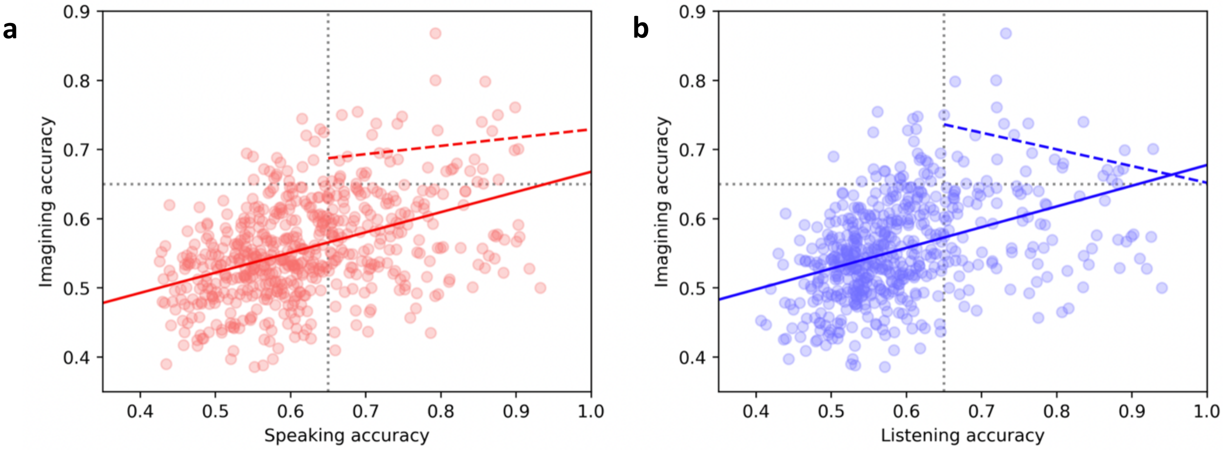

*Supplementary Figure 2 – **Relation between speech mode accuracies**. Imagined speech accuracy is plotted as a function of the performed (panel **a**) and perceived (panel **b**) speech accuracy. Each dot corresponds to an electrode (all electrodes from all participants were included in this analysis). The solid lines depict the regression lines across all points (correlation of 0.4253 and 0.4086 for panel a and b, respectively), while the dashed lines depict the regression lines for electrodes whose accuracies for both speech modes are above 65% (correlation of 0.1975 and -0.3730 for panel a and b, respectively).*

Supplement to Figure 3

	Speaking	Listening	Imagining
P1	3.59e-02	3.33e-02	9.13e-03
P2	8.04e-01	9.05e-01	6.05e-01
P3	4.85e-02	1.37e-03	8.02e-02
P4	8.16e-04	2.97e-02	1.85e-01
P5	7.33e-02	2.97e-02	5.08e-01
P6	1.66e-02	4.47e-02	2.42e-01
P7	8.61e-01	1.00e+00	1.00e+00
P8	4.86e-03	3.68e-02	4.01e-01
P9	4.42e-04	3.39e-02	3.08e-01
P10	3.67e-02	2.53e-02	3.38e-01
P11	9.03e-02	3.11e-02	2.53e-02
P12	2.25e-01	4.30e-01	5.12e-01
P13	6.92e-01	8.82e-01	5.67e-01
P14	1.81e-02	1.74e-03	3.34e-01
P15	3.79e-02	-	3.58e-01
P16	1.71e-02	2.20e-01	1.83e-01

*Supplementary Table 6 – **Single- vs multi-electrode models comparison**. P-values for the statistical test (one-sided Wilcoxon signed-rank test) comparing the trial accuracies for the optimal multi-electrode model with the best single-electrode model.*

Description of Supplementary Fig. 3 and 4

For each participant, the performance for each modality is depicted for both single- and multi-electrode models in the left panel. Each violin plot depicts the distribution of trial accuracies. The middle line segment depicts the mean while the upper and lower line segments depict the extrema. The asterisk indicates whether the multi-electrode model outperforms the single-electrode model (* p-value < 0.05, one-sided Wilcoxon signed-rank test, see Supplementary Table 6). The optimal number of electrodes is indicated below the multi-electrode performance. The right panel depicts the accuracy plots for an increasing number of electrodes for each speech mode. The dotted line depicts the accuracy of the best single-electrode model. Black asterisks indicate when the statistical test was significant ($p<0.05$) through the iterative process and colored asterisks indicate the optimal multi-electrode models. A dot indicates the optimal model if no multi-electrode model performed significantly better than the single-electrode model. The number of electrodes of the optimal multi-electrode model is indicated on top of each plot.

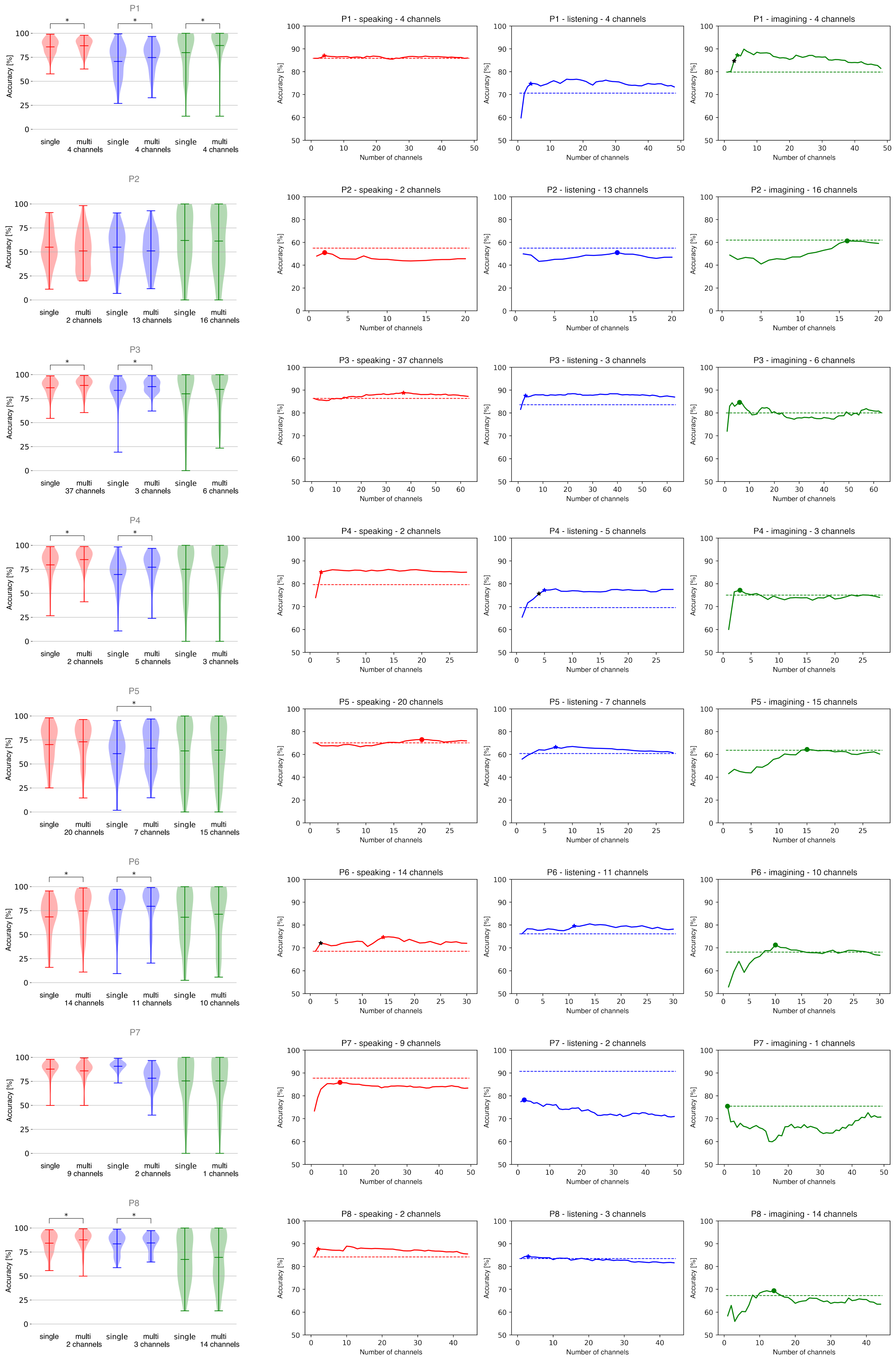

Supplementary Figure 3 – see description above

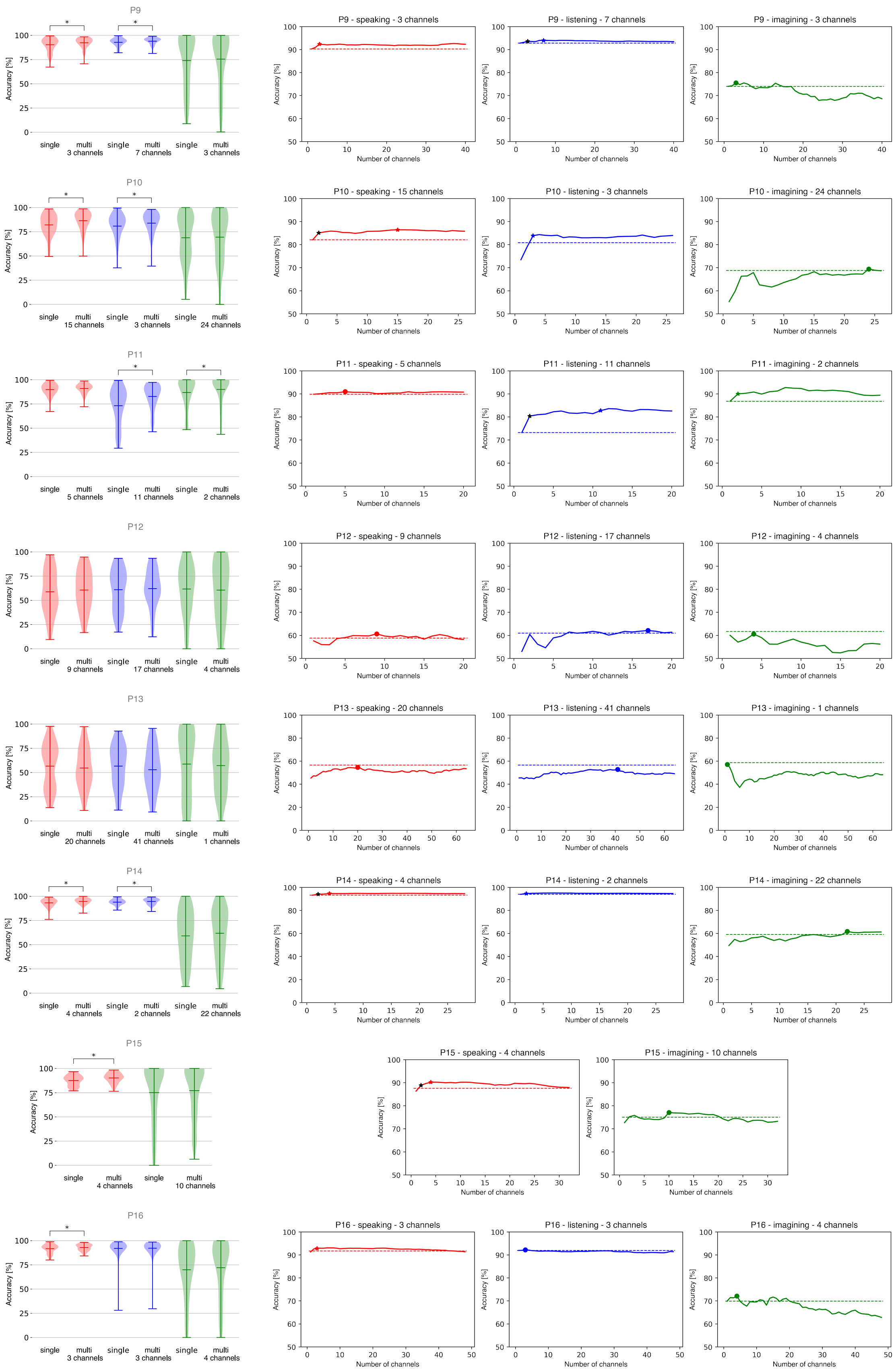

Supplementary Figure 4 – see description above

Supplement to Figure 4

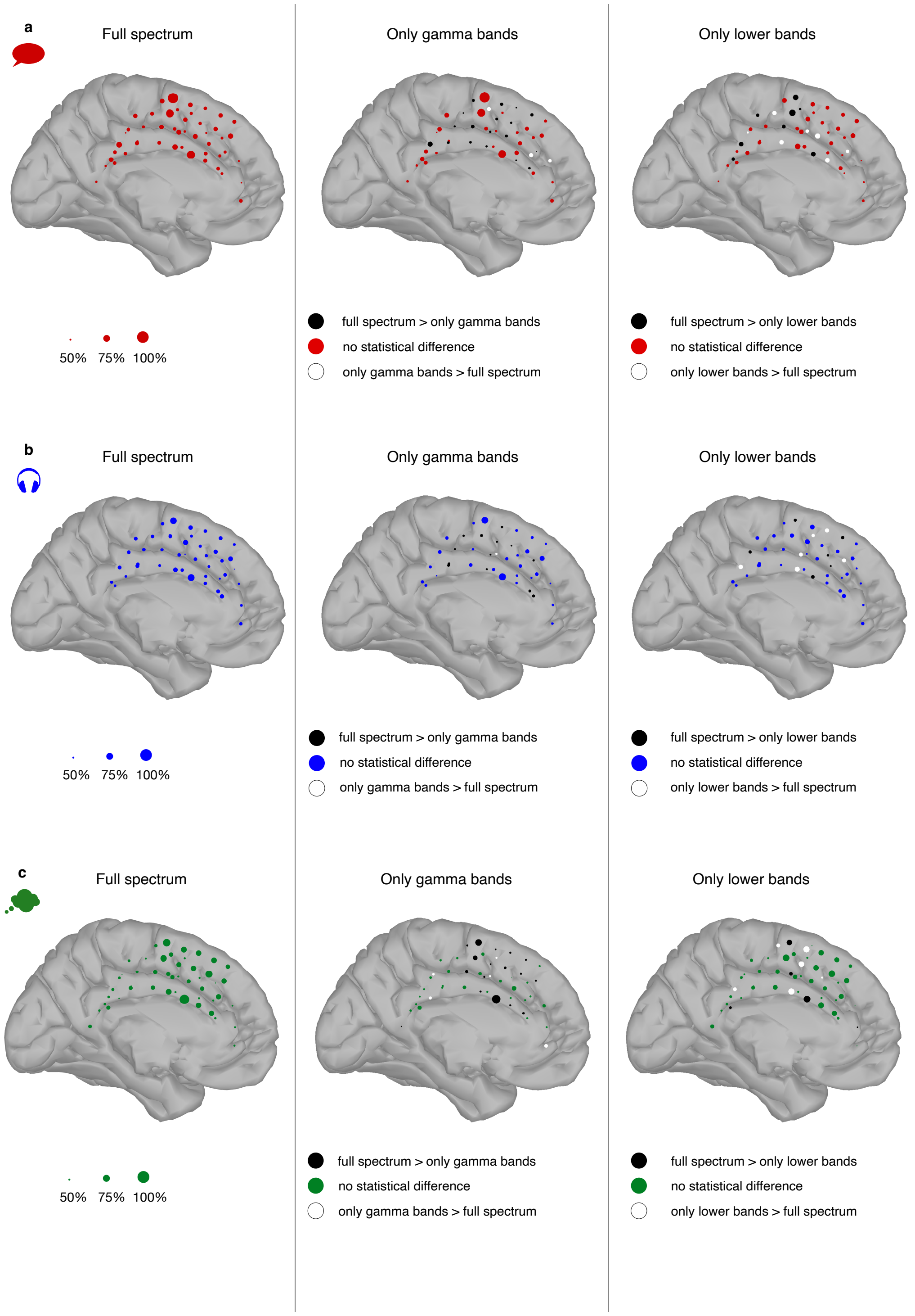

*Supplementary Figure 5 – **Medial views of Figure 4.***

Supplement to Figure 5

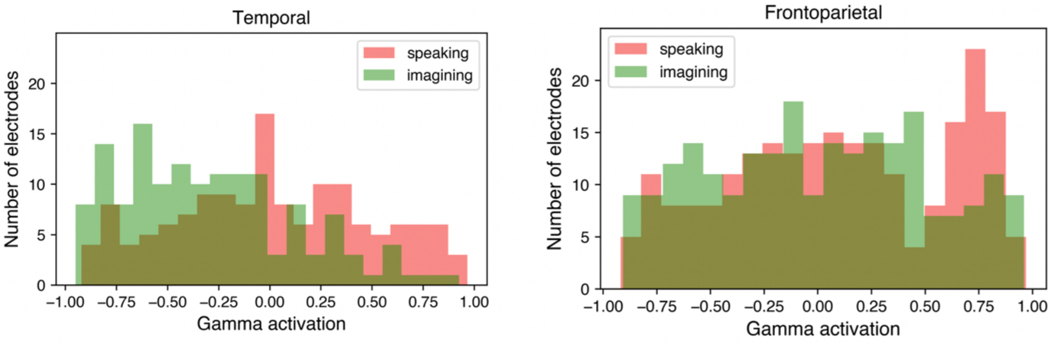

*Supplementary Figure 6 – **Gamma activation comparison**. Distribution of gamma activation across the electrodes for performed and imagined speech in the temporal lobe and the frontoparietal region. Electrodes from the 10 participants with at least one electrode performing better than chance level for imagined speech detection were included (143 electrodes in temporal lobes and 232 electrodes in the frontoparietal region). The difference between the gamma activation of performed and imagined speech was tested in both the temporal lobe and the frontoparietal region (Wilcoxon signed-rank test, alternative: gamma activation of imagined speech < performed speech, p= 1.42e-09 and p= 0.0377, respectively).*

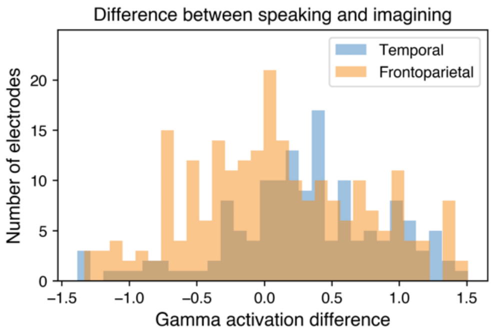

*Supplementary Figure 7 – **Gamma activation difference between speaking and imagining**. Distribution of the difference between imagined and performed speech across electrodes in the temporal lobe and frontoparietal region. Electrodes from the 10 participants with at least one electrode performing better than chance level for imagined speech detection were included (143 electrodes in temporal lobes and 232 electrodes in the frontoparietal region). The difference was found to be larger in the temporal lobe than in the frontoparietal region (Wilcoxon rank-sum test, p=1.29e-04, alternative: difference in the temporal lobe > difference in the frontoparietal region).*

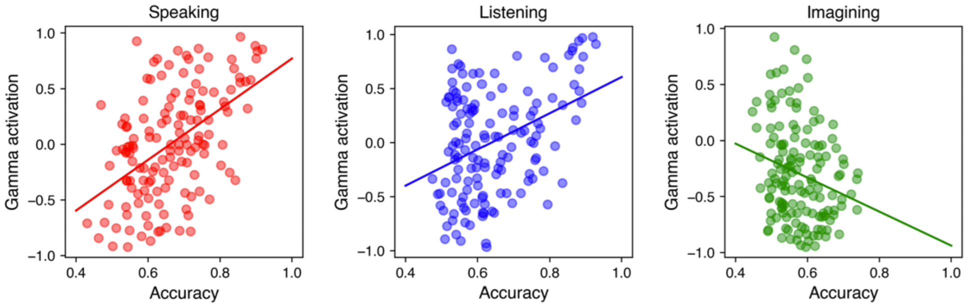

*Supplementary Figure 8 – **Relation between gamma activation and model accuracy in the temporal lobe**. Each dot corresponds to an electrode in the temporal lobe. Electrodes from the 10 participants with at least one electrode performing better than chance level for imagined speech detection were included (143 electrodes in temporal lobes). The line depicts the regression line. Correlation values were 0.5078, 0.3894 and -0.2340 for performed, perceived and imagined speech respectively.*

Supplement to Figure 6

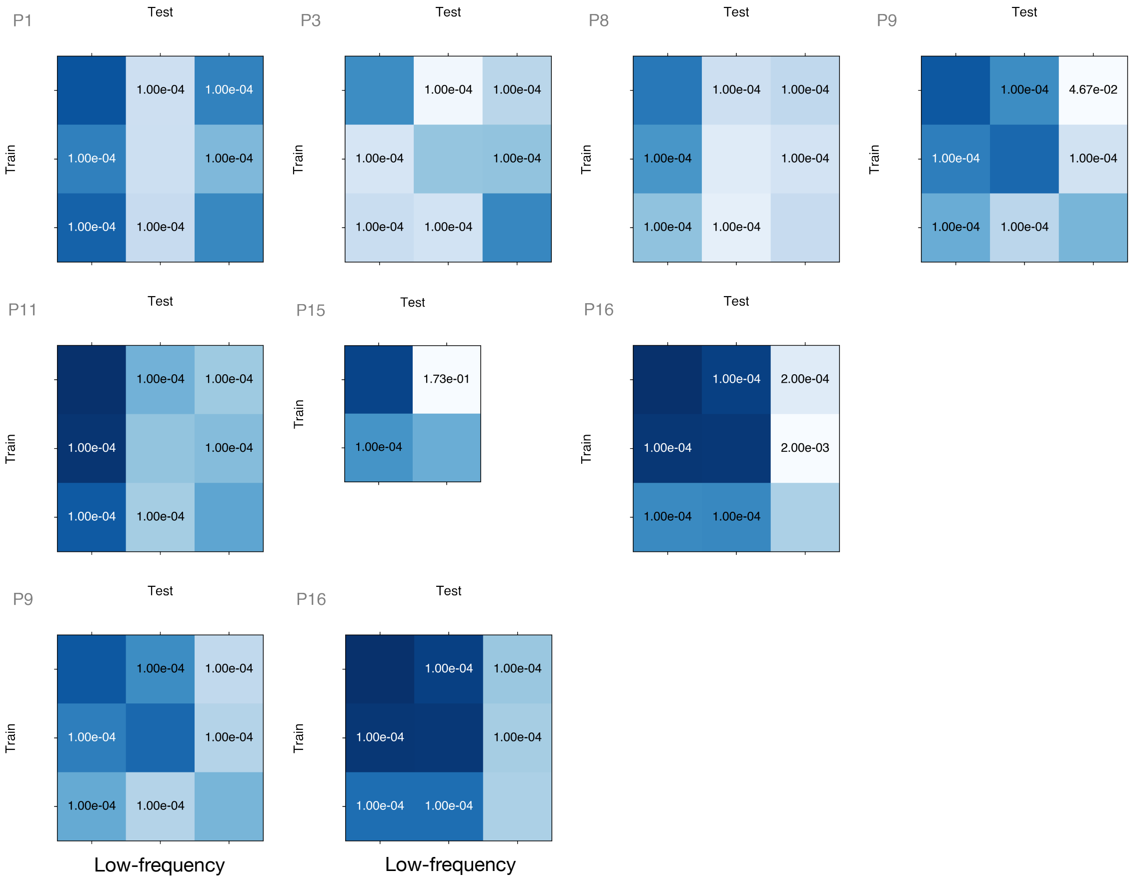

*Supplementary Figure 9 – **Model transfer across speech modes statistics.** P-values for the statistical test (permutation test) for the model transfer across speech modes.*

Supplement to Figure 7

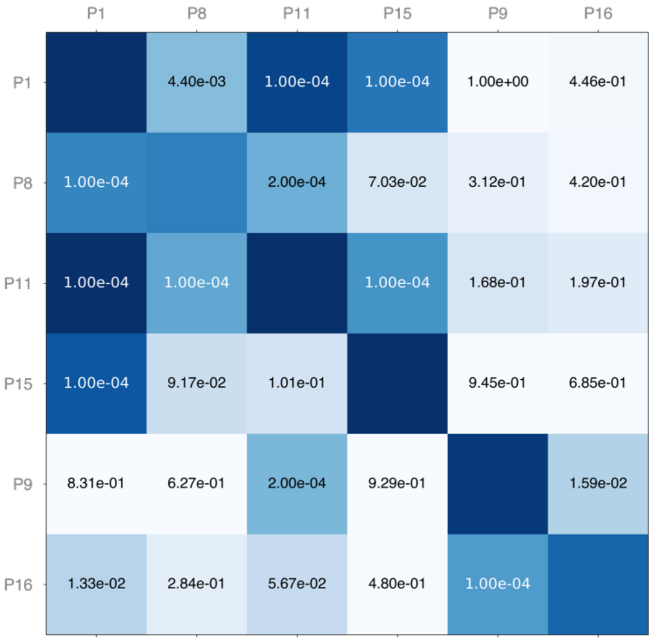

*Supplementary Figure 10 – **Model transfer across participants statistics.** P-values for the statistical test (permutation test) for the model transfer across participants.*

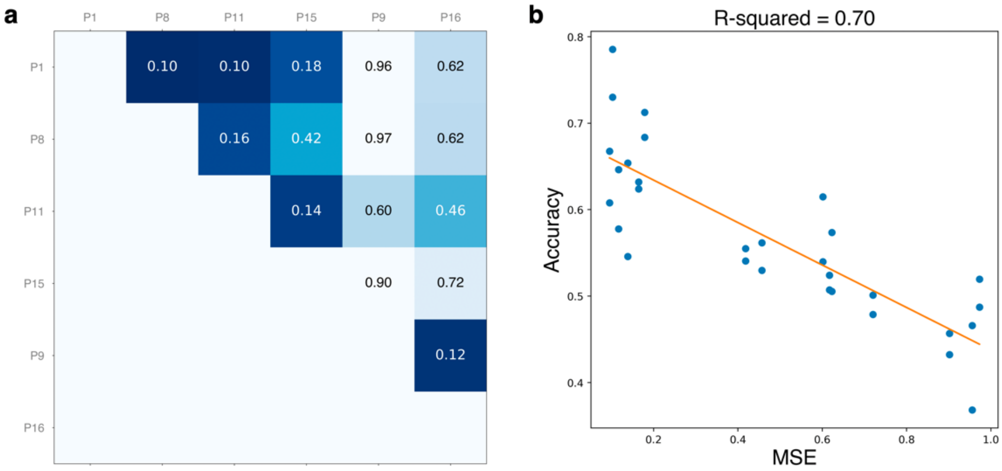

*Supplementary Figure 11 – **Comparison between transfer performance and activation coefficients distance**. **a** The mean square error (MSE) between subjects' activation patterns is shown. **b** The performance of the model transfer (in terms of accuracy) was compared to the distance between the activation patterns. Blue dots correspond to compared subjects with the regression line marked in orange (r-squared value shown on top).*

	Participant	Individual models	Mean	One model for all	P-value	Mean
Motor	P1	79.83%	74.57%	80.84%	0.8282	71.34%
	P8	67.29%		65.78%	0.2769	
	P11	76.13%		76.68%	0.3827	
	P15	75.03%		62.07%	0.0688	
Temporal	P9	74.01%	71.96%	77.04%	0.8426	72.77%
	P16	69.91%		68.49%	0.2232	

Supplementary Table 7 – ***Multiple-subjects model performance.*** *For each subject, the performance of the individual model is compared to the model using multiple subjects. The p-value of the statistical test is shown (one-sided Wilcoxon signed-rank test, null hypothesis: trial accuracies with the individual model are greater than with the population model).*
