# Supplementary material for: Imagined speech event detection from electrocorticography and its transfer between speech modes and subjects
Source: Commun Biol. 2024 Jul 5;7:818. doi: 10.1038/s42003-024-06518-6 (PMC11226700; doi:10.1038/s42003-024-06518-6)
Supplement: Supplementary file 3 — Description of Additional Supplementary Files [file 42003_2024_6518_MOESM3_ESM.pdf]

Description of Additional Supplementary Files

File name: Supplementary Data 1

Description: Excel files with the data used for Fig. 2 and 4. For each electrode, the accuracy is given for each speech mode with the model trained with the full spectrum, only gamma bands and only lower bands.

File name: Supplementary Data 2

Description: Excel files with the data used for Fig. 3 and Supplementary Fig. 3 and 4. For each trial (leave-one-out cross-validation), the accuracy is given for each speech mode with the single-electrode and multi-electrode model.
