# Supplementary material for: Imagined speech event detection from electrocorticography and its transfer between speech modes and subjects
Source: Commun Biol. 2024 Jul 5;7:818. doi: 10.1038/s42003-024-06518-6 (PMC11226700; doi:10.1038/s42003-024-06518-6)
Supplement: Supplementary file 6 — Reporting summary [file 42003_2024_6518_MOESM6_ESM.pdf]

nature portfolio

Corresponding author(s): DAPR

Last updated by author(s): May 30, 2024

Reporting Summary

Nature Portfolio wishes to improve the reproducibility of the work that we publish. This form provides structure for consistency and transparency in reporting. For further information on Nature Portfolio policies, see our Editorial Policies and the Editorial Policy Checklist.

Statistics

For all statistical analyses, confirm that the following items are present in the figure legend, table legend, main text, or Methods section.

n/a	Confirmed	
☐	☒	The exact sample size (*n*) for each experimental group/condition, given as a discrete number and unit of measurement
☐	☒	A statement on whether measurements were taken from distinct samples or whether the same sample was measured repeatedly
☐	☒	The statistical test(s) used AND whether they are one- or two-sided<br>*Only common tests should be described solely by name; describe more complex techniques in the Methods section.*
☒	☐	A description of all covariates tested
☒	☐	A description of any assumptions or corrections, such as tests of normality and adjustment for multiple comparisons
☐	☒	A full description of the statistical parameters including central tendency (e.g. means) or other basic estimates (e.g. regression coefficient) AND variation (e.g. standard deviation) or associated estimates of uncertainty (e.g. confidence intervals)
☐	☒	For null hypothesis testing, the test statistic (e.g. *F*, *t*, *r*) with confidence intervals, effect sizes, degrees of freedom and *P* value noted<br>*Give P values as exact values whenever suitable.*
☒	☐	For Bayesian analysis, information on the choice of priors and Markov chain Monte Carlo settings
☒	☐	For hierarchical and complex designs, identification of the appropriate level for tests and full reporting of outcomes
☐	☒	Estimates of effect sizes (e.g. Cohen's *d*, Pearson's *r*), indicating how they were calculated

Our web collection on statistics for biologists contains articles on many of the points above.

Software and code

Policy information about availability of computer code

Data collection	Brain data was recorded using UZ Gent’s SD LTM 64 Express amplifier (Micromed, Italy) and audio data using an AT875R directional microphone. Stimulus presentation was implemented with Psychtoolbox-3 in Matlab R2017.
Data analysis	Data was analyzed in Matlab R2021b and Python 3.9.7.

For manuscripts utilizing custom algorithms or software that are central to the research but not yet described in published literature, software must be made available to editors and reviewers. We strongly encourage code deposition in a community repository (e.g. GitHub). See the Nature Portfolio guidelines for submitting code & software for further information.

Data

Policy information about availability of data

All manuscripts must include a data availability statement. This statement should provide the following information, where applicable:

- Accession codes, unique identifiers, or web links for publicly available datasets
- A description of any restrictions on data availability
- For clinical datasets or third party data, please ensure that the statement adheres to our policy

The datasets presented in this article are not readily available because of the sensitive nature of intracranial patient data. Requests to access the datasets should be directed to the corresponding author.

Research involving human participants, their data, or biological material

Policy information about studies with human participants or human data. See also policy information about sex, gender (identity/presentation), and sexual orientation and race, ethnicity and racism.

Reporting on sex and gender	Sex is reported in the manuscript.
Reporting on race, ethnicity, or other socially relevant groupings	This information has not been collected since it was not relevant to our study.
Population characteristics	Age is reported in the manuscript. All patients suffer from drug-resistant epilepsy.
Recruitment	The participants are epilepsy patients temporarily implanted with ECoG electrodes as part of their clinical workup. Every Dutch-speaking patient with an implant over the temporal, frontal and/or parietal lobes was proposed to participate in the study. Patients who agreed to participate signed an informed consent form.
Ethics oversight	KU Leuven and Ghent University Hospital

Note that full information on the approval of the study protocol must also be provided in the manuscript.

Field-specific reporting

Please select the one below that is the best fit for your research. If you are not sure, read the appropriate sections before making your selection.

☒ Life sciences ☐ Behavioural & social sciences ☐ Ecological, evolutionary & environmental sciences

For a reference copy of the document with all sections, see nature.com/documents/nr-reporting-summary-flat.pdf

Life sciences study design

All studies must disclose on these points even when the disclosure is negative.

Sample size	No sample-size calculation was performed. All subjects that took part in the study are reported (19 subjects).
Data exclusions	3 participants reported they could not imagine speaking and were excluded from the data analysis (reported in the Methods section).
Replication	Data from 16 subjects was analyzed, which is a relatively large dataset for ECoG recordings. We found significant trends across patients.
Randomization	No allocation in groups
Blinding	No allocation in groups. The performance of the models was assessed through a cross-validation procedure.

Reporting for specific materials, systems and methods

We require information from authors about some types of materials, experimental systems and methods used in many studies. Here, indicate whether each material, system or method listed is relevant to your study. If you are not sure if a list item applies to your research, read the appropriate section before selecting a response.

Materials & experimental systems

n/a	Involved in the study	
☒	☐	Antibodies
☒	☐	Eukaryotic cell lines
☒	☐	Palaeontology and archaeology
☒	☐	Animals and other organisms
☒	☐	Clinical data
☒	☐	Dual use research of concern
☒	☐	Plants

Methods

n/a	Involved in the study	
☒	☐	ChIP-seq
☒	☐	Flow cytometry
☒	☐	MRI-based neuroimaging
